# Psychological experiences and needs of tumor patients with implanted intravenous infusion ports: a qualitative study

**DOI:** 10.3389/fonc.2024.1392416

**Published:** 2024-05-16

**Authors:** Lan Zhu, Kun Li, Qiu He, Lin Liu

**Affiliations:** ^1^1Department of Nursing, Jiangxi Provincial People’s Hospital ,The First Affiliated Hospital of Nanchang Medical Collage, Nanchang, Jiangxi, China; ^2^Department of Neurology, Nanchang People’s Hospital, Nanchang, Jiangxi, China; ^3^Department of Nursing, The Second Xiangya Hospital, Central South University, Changsha, Hunan, China

**Keywords:** cancer, IVAP, psychology, needs, qualitative research

## Abstract

**Background:**

There are many problems of psychological burden in patients with tumor implanted in port of intravenous infusion. However, more attention is paid to its complications in the literature, and psychological problems are seldom concerned. The purpose of this study was to explore the psychological state and needs of tumor patients after implantation of an intravenous infusion port and provide valuable references for psychological interventions.

**Method:**

A semi-structured interview was conducted with 11 patients with intravenous infusion ports. Colaizzi’s 7-step analysis was used to analyze the interview data.

**Results:**

According to the primary information, four themes and nine sub-themes were extracted: (1) lack of self-worth, (2) multiple emotional experiences (guilt, doubt, worry, and gain). (3) Poor self-management and self-maintenance awareness (over-reliance on medical staff, unchanged family roles, lack of related knowledge). (4) Expectations and suggestions for the future (inner expectations, suggestions for infusion ports).

**Conclusion:**

The patient’s psychological state should be carefully monitored during tube implantation, to relieve the patient’s tension and anxiety and improve nursing satisfaction and patient outcomes.

## 1 Introduction

With the transformation of the biomedical model and disease spectrum, malignant tumor has become the second major disease that threatens human life at present (1). The latest figures show that as of 2022, there are about 4.83million new cases of malignant tumors and 2.58million deaths in China, with a diverse range of new tumor types and a further exacerbation of the cancer burden, a serious threat to people’s life and health security (2). At present, malignant tumors are treated primarily with surgery, chemotherapy, radiotherapy, and immunotherapy. Chemotherapy is an essential component of the comprehensive treatment of malignant tumors. Oral, intravenous, and intraperitoneal chemotherapy are the most common routes of chemotherapy, and intravenous chemotherapy is the most common way. Traditional intravenous drug administration can cause many kinds of discomfort, which directly affects the clinical treatment outcome and reduces the quality of life of patients (3).

*Implanted Venous Access Port* (IVAP) as the latest infusion pathway solves the above problems. It is mainly used in cancer patients (4–6). The port is a closed venous infusion system with a port body attached to a central venous catheter under the skin. The internal jugular and the subclavian veins are the main routes and are surgically placed (7). Infusion ports can effectively alleviate the pain caused by frequent replacement of infusion lines (8). Tumor patients in the infusion port indwelling period, general maintenance every four weeks once, to patients with convenience. Compared with other venipuncture tools, it has many advantages, such as being easy to carry, requiring less repeated puncture, and having a long indwelling time. It is considered an ideal channel for intravenous chemotherapy in cancer patients (9, 10).

However, patients in IVAP will face many problems during the intermission period of chemotherapy. The risk of complications related to the infusion port will increase after leaving the hospital for a long time under the guidance of specialized nurses in the infusion port. It affects the normal course of treatment and the continuous use of infusion ports, which causes significant pain to patients (11). Pan et al. (12) analyzed the clinical infection complication and infection rates of IVAP in solid tumor chemotherapy patients and found that 8 of 495 patients developed infections within 30 days after the operation (early infection). Thirty-one patients developed late infection 30 days after the operation. Research has found that (13), the part of the catheter in the infusion port caused by mopping the floor at home, the obstruction of the catheter caused by the increased pressure of the chest caused by long-term constipation, the skin infection around the port caused by not bathing for a long time, and the failure to come to the hospital on time all hindered the chemotherapy in the infusion port to some extent, causing the patient considerable inconvenience. The main reasons were that most patients lacked self-management awareness, ability, self-persistence, confidence, and family support. Therefore, improving the self-care of IVAP patients, bolstering their enthusiasm for home maintenance and communicating with medical staff are crucial. However, the current focus is on the clinical application of infusion port and related studies of complications (14, 15). Few studies have been conducted on patients’ psychological states during the period of catheterization in transfusion ports. Therefore, this study explored patients’ actual feelings and needs during the period of catheterization through semi-structured interviews and provided references for clinical intervention.

## 2 Methods

This study used the phenomenological method of qualitative research and semi-structured interviews to understand the psychological experience of IVAP patients with cancer in a top-three hospital in Hunan province, China. This study was approved by the Hunan Normal University Ethics Committee.

### 2.1 Participants and sample size

The inclusion criteria were: (1) patients with a clinical pathology diagnosis of malignancy, (2) patients undergoing chemotherapy and using an IVAP, (3) patients with IVAP for more than one month, (4) age ≥18 years, (5) informed consent and volunteering to participate in this study.

The exclusion criteria were: (1) patients with other serious diseases, (2) patients with mental disorders or psychiatric history, and (3) those who could not participate in the study due to other reasons.

### 2.2 Recruitment process

According to the inclusion and exclusion criteria, the appropriate sample size was selected purposefully. The study was conducted with the consent of the hospital and the subjects. The principle of determining the sample size is that no new information appears after selecting new samples. After interviewing the 11th subject in this study, the information reached saturation. Therefore, 11 patients participated in the interview. general information about the interviewees in **Table 1**.

**Table 1:** General patient information.

Serial number	age	Education level	Marital status	Employment status	Average monthly household income(RMB)	Primary caregiver	Complications or not
A1	61	Junior High School	Married	Unemployed	1001–3000	Children	No
A2	35	High School or technical secondary school	Married	Unemployed	3001–5000	Spouse	No
A3	65	Primary and below	Married	Unemployed	1001–3000	Children	Yes
A4	42	University and above	Divorce	On the job	3001–5000	Brothers and sisters	No
A5	44	High School or technical secondary school	Married	Unemployed	3001–5000	Spouse	No
A6	35	High School or technical secondary school	Married	On the job	1001–3000	Spouse	No
A7	42	High School or technical secondary school	Married	Unemployed	≥5000	Parents	No
A8	55	Primary and below	Married	Unemployed	1001–3000	Children	No
A9	51	Junior High School	Married	Retirement	1001–3000	Children	Yes
A10	58	Primary and below	Married	Unemployed	3001–5000	Children	No
A11	53	Primary and below	Married	Unemployed	≤1000	Spouse	Yes

### 2.3 Data collection

The interviews were conducted face-to-face, and informed consent was obtained and signed before the interview. The interview was conducted in a quiet conference room or ward, avoiding the treatment time. The interview time was about 1 hour. During the interview, the subjects were recorded on their cell phones, and their movements and expressions were recorded with pen and paper, encouraging them to express their true feelings as much as possible. See **Table 2** for the interview outline:

**Table 2:** Interview outline.

1.Why do you choose to install intravenous port, mainly out of which aspects of the considerations?
2.What do you know about IVAP? Where did you get the relevant knowledge?
3.Have Your Life and work been affected by IVAP? How do you deal with these effects?
4.What are your thoughts and feelings about IVAP?
5.Have you had any problems with the tube? What did you do then?
6.What kind of help do you expect from your health care provider in relation to the infusion port?
7.When choosing to install the infusion port, what was your family’s attitude at that time?
8.What is the physical, work and emotional state of your family taking care of you while you are using IVAP?
9.Does your family offer you any help or support? What is your greatest feeling about your family’s care?
10.Are you feeling stressed right now? What’s the main reason and how do you usually deal with it?
11.What are you most worried about right now What are your needs and suggestions?

### 2.4 Data analysis

The recording was translated into text within 24 hours of the interview. The information was promptly clarified with the interviewees if the recording was unclear to ensure its authenticity and comprehensiveness. The data were collected and analyzed simultaneously. The data were then analyzed and arranged according to Colaizzi’s phenomenological seven-step analysis method (16) to extract critical and essential information.

### 2.5 Quality control

Substantial literature was reviewed, and relevant experts were consulted to construct an interview outline. After the pre-interview, the outline was reviewed to make appropriate adjustments to understand patients’ actual experiences. Within 24 hours of the two interview’s conclusion, the recorded information was converted into written information. This step facilitated timely inquiry and confirmation so that the information obtained was reliable.

## 3 Results

### 3.1 Lack of self-worth

Generally speaking, the realization of patients’ self-worth is mainly reflected in the ability to assume the corresponding responsibilities and obligations, achieve their own life goals, return to society, and confront other challenges. However, after the patient was implanted with an intravenous port, his life and work were also affected to a certain extent. In many cases, he could only choose to rest at home and could not return to his job, a reluctance to socialize, travel, and engage in other activities.

“*I look like this sometimes feel inferior, the crowd I do not go, a person to go for a walk, hair also cut, afraid of others look down on me (tears)* ” (A1)

“*I usually will pay attention to (infusion port), now is not doing anything, class is not on, wait for good again*” (A4)

“*Now I am also temporarily off work, then will consider quitting it, or to rest oriented*” (A6)

### 3.2 Multiple emotional experiences

**(1) Guilt**

Patients, especially after implantation of the intravenous port, showed a certain degree of guilt that the long-term treatment and care for the family had brought with it specific burdens, which will reduce their compliance, failure to cooperate with treatment or regular maintenance of infusion port.

“*My daughter said to give me money, I am not willing to let her out, she also has their own family, husband, children, I am afraid of other people conflict, I would rather suffer some losses, also do not want to see them conflict*” (A1)

“*Now back at home is mainly my mother to take care of me, she is more than seventy years old, the body is not too good, I still feel a little sorry*.” (A5)

“*Think I actually state of mind is not very good, I will be very nervous, but generally will hide the family, I do not want to let the family worry (choking)*” (A7)

“*Sometimes think of treatment to use a lot of money, I feel uncomfortable, other people’s parents will help their children, I have to implicate them*” (A8)

**(2) Doubt**

The patients were unfamiliar with IVAP, and some were confused during the extubation period. They will worry about whether the infusion port will bring harm to themselves, whether it will affect their lives or work, and their financial ability to afford, and so on.

“*After the chemo, my port of infusion will be pulled out, Did the extubation take long ? Will there be adverse reactions?*” (A3)

“*After the first chemotherapy, there was a sense of soreness and distension in the grip of the ball. It would be better if I didn’t pinch it. I also didn’t know how much force the ball should hold*” (A4)

“*Always think of if there is a problem in the infusion port, the drug contact with our body, will it cause ulceration ah, do not know the safety of this infusion port*” (A7)

**(3) Worry**

Although most patients believed that intravenous ports did not affect daily life, some reported having various concerns.

“*There is a three-year-old child at home, always worried about what will cause problems, so they dare not hug her*.” (A2)

“*At the beginning of the port is the main infusion of fear of thrombosis, after all, this is more serious*.” (A4)

“*Take a bath choose Sitz Bath, dare not shower. Very worried, dare not to use water to flush*.” (A5)

“*Still quite worried, afraid of infusion port in my body can be broken how to do*.” (A6)

Some patients also said that the port of intravenous infusion impacted their self-images. Moreover, they were concerned about others’ evaluation of them.

“*Fortunately, the clothes covered [me], others cannot see*.” (A2)

“*When I wear clothes in the summer, I still choose to wear taller clothes to cover, outside will be afraid of being seen*” (A5)

**(4) A sense of benefit**

IVAP also benefited some patients. After making a comparison, they chose to use the infusion port, believing that it was convenient and further improved their quality of life.

“*Before I used the PICC, [I] found that the port infusion or a lot of benefits, do not have to go to the hospital every week maintenance, winter clothing is also convenient*.” (A3)

“*The choice of infusion port is certainly less harmful to patients, but also convenient*.” (A8)

### 3.3 Weakened sense of self-management and self-maintenance

**(1) Over-reliance on medical staff**

Some patients fully acknowledged and even felt dependent on nursing support. They believe that any problems with the hospital to help on the line, while ignoring the impor- tance of the infusion port self-management.

“*We do what the nurses tell us. We don’t know anything*.” (A8)

“*After implantation port, think there is nothing to pay attention to, to the hospital on it, the doctors and nurses will tell us what to do, we do not care*.” (A9)

**(2) The role of the family has not changed**

Most women were the primary providers and caregivers for family members. However, they did not adjust their roles after the infusion port implantation. They were afraid of hindering other family members.

“*Nothing has changed in my life. I still do the same housework. Anyway, don’t treat yourself as a patient, do what you can do*.” (A2)

“*I can’t do anything with PICC, so I prefer to use the infusion port. I have old people and children to take care of at home. I can’t let them take care of me*.” (A6)

**(3) Lack of relevant knowledge**

During the interview, patients described various daily life problems. They did not know how to manage them due to their lack of knowledge.

“*Nurse sent me a study manual for the tube, and I didn’t read it*.” (A8)

“*I didn’t know that I had to maintain every month without chemotherapy*.” (A10)

“*I’m allergic to this tape. It itches, but I don’t know what to do, so I tear it off*.” (A11)

### 3.4 Expectations and suggestions for the future

**(1) Expectations**

Disease, chemotherapy, and infusion ports were accompanied by patient pain and anxiety. Patients’ conditions, family situations, and future expectations were altered.

“*Now I have some phobia about the infusion port, and I hope to pull it out as soon as I finish the chemotherapy*.” (A3)

“*I am divorced, now my biggest pressure, the child is still young, I must treat, watching her grow up*.” (A4)

“*Hope to get well soon, want to go home, here every day to see strangers, no friends. (Bow)* ” (A10)

**(2) Advice to IVAP**

While acknowledging the need for the intravenous infusion port, the patient also pointed out its deficiencies and areas needing improvement.

“*This infusion port is very good, but the time is long, the old people’s body cannot take it. Hope to improve in the future, to ease the pain of patients*.” (A3)

“*I am allergic to some of the film paste, very itchy, scratch broken if you can improve it*.” (A5)

“*I am allergic to the adhesive tape. I had to tear it off in five days. I tore it off in two days. I just hope the medical staff will tell us more things that we need to pay attention to*.” (A7)

## 4 Discussion

In this study, their education level is not high, in this kind of cultural background, They are not interested in learning, so the new infusion tool (IVAP) will generate more anxiety and anxiety. In addition, many of them are unemployed, and not having a job means they may not be able to live up to some of their values, and their beauty is affected by the port, and they don’t want to socialize, the lack of Self-worth is easy to appear. They also need the care of their children or spouses, and the psychological guilt may affect the treatment of the disease and the nursing of the infusion port to some extent.

Intravenous port is more convenient and comfortable than PICC, it has many advantages. This study is more concerned about the psychological needs of patients, so that medical care more from the point of view of patients, to solve more problems for them. The four themes extracted from this study also give us some enlightenment. First, we should draw up a scientific and detailed evidence-based practice plan for our country, such as the prevention of complications and self-care, so as to better guide patients; Secondly, in the process of using the port, we should strengthen the training of related knowledge, and solve the patients’ questions by reproducing real cases and teaching videos, so as to promote the patients’ better recovery; In addition, it is essential to actively improve continuous care, although self-management ability of infusion port is very important, but many professional problems patients cannot solve, the nurse should take the initiative to solve the patient’s problems regularly after discharge, and carry out home nursing if necessary; In addition, it is worth mentioning that IVAP are more expensive than PICC, and more health insurance policies are needed to alleviate the economic pressure on patients.

This study showed that patients experience a diminished sense of self-worth. They readily lose their self-confidence, become avoidant, and even report suicidal ideation. According to Maslow’s hierarchy of needs, self-actualization is a basic need that enhances the experience of happiness. Therefore, bolstering the self-worth of patients with tumor implantations is crucial. These patients feel inferior in daily life, social life, and work because of infusion ports. Medical staff should take intervention measures according to different patients’ conditions to enhance their self-worth (17, 18). One way to accomplish this goal would be through peer support for education, asking other patients who have the same experience to provide information and emotional support (19, 20). However, family members should also be encouraged to participate and provide patients with more support and understanding. For example, cancer patients are unable to do much housework after the intravenous infusion port, with a corresponding reduction in cost to the family. Family care is critical at this time. Along with treatment and nursing care, family support also helps patients regain self-confidence and enhances their self-worth.

This study showed that patients had multiple emotional experiences after infusion port implantation, mainly manifested in patients’ guilt, uncertainty, and worry. As an infusion tool, the infusion port has brought many advantages, such as convenience to the patients as far as possible. Still, at the same time, it has also caused some anxiety for patients and increased their psychological burden to some extent. In addition, after diagnosis, patients generally worry about the effects of treatment and the infusion port caused by physical discomfort and self-image disorder (21–23). Patients believe that the infusion port is a “foreign body,” and will interfere with their lives. They also worry that the infusion port will affect their attractiveness (24). In addition, patients depend on family care but also often feel guilty about such dependence. Notably, a significant proportion of patients also believe that infusion port improves their quality of life because it is more convenient than PICC. It has lower complication rates and produces higher overall satisfaction (25–27). Therefore, we must proceed optimistically, working to enhance patients’ positive experiences with the infusion port and reduce their emotional distress.

Improving patient self-management helps to extend infusion port life and reduce costs (28, 29). This study found that a weak sense of self-management relates to the following aspects. First, the patient over-depends on the medical staff, thinking if doctors, nurses, and or other healthcare staff are available, they have no reason for concern. Second, the patient’s role within the family should change. After the vein infusion port is implanted, the patient should limit heavy physical activity, such as frequent floor-mopping and other labor-intensive household tasks. However, patients worry about the burden on their families. They tried to do their share of household work and did not make appropriate changes. Third, they lacked relevant professional knowledge. In the daily self-maintenance process, patients were often at a loss when confronted with problems or even ignored them. Therefore, nurses should actively strive to provide excellent health education. They should devise various forms of instruction, for example, regular trainings or lectures for patients, making short videos or creating knowledge manuals to foster patients learning as outpatients. Moreover, they should provide regular telephone reminders and online answers to patient questions. These strategies will help the patient grasp the importance of IVAP and understand how it works.

## 5 Limitations

This study aimed to explore patients’ psychological changes and needs after catheterization. However, it did not investigate their inner state before this intervention. Only eleven participants were interviewed in this study, which limits the generalization of its results. Larger sample and multi-center studies should be carried out in the future to enhance and support this study’s findings.

## 6 Conclusion

This study conducted in-depth interviews to explore the psychological state of 11 patients with tumor implantations at intravenous infusion ports. Four themes and nine sub-themes were extracted, including diminished sense of self-worth, various emotional experiences during placement of the tube, limited awareness of self-management and self-maintenance, and expectations and suggestions for the future. Through understanding the psychological process of the patients during the period of tube-carrying, the findings suggest that nurses should pay attention to the different inner experiences of patients after tube implantation, strengthen compassionate care and health education, and reduce emotional distress. Moreover, they suggest the need for further exploration of the infusion port’s benefits to improve patient satisfaction and outcomes.

## Data availability statement

The original contributions presented in the study are included in the article/supplementary material. Further inquiries can be directed to the corresponding author.

## Ethics statement

The studies involving humans were approved by the Hunan Normal University Ethics Committee. The studies were conducted in accordance with the local legislation and institutional requirements. The participants provided their written informed consent to participate in this study.

## Author contributions

LZ: Writing – original draft, Resources, Investigation. LL: Writing – review & editing, Supervision, Formal analysis. KL: Writing – review & editing, Data curation. QH: Writing – original draft, Data curation.
